# Policy Inertia on Regulating Food Marketing to Children: A Case Study of Malaysia

**DOI:** 10.3390/ijerph18189607

**Published:** 2021-09-12

**Authors:** SeeHoe Ng, Bridget Kelly, Heather Yeatman, Boyd Swinburn, Tilakavati Karupaiah

**Affiliations:** 1 Early Start, School of Health and Society, University of Wollongong, Wollongong, NSW 2522, Australia; seehoe88@gmail.com or bkelly@uow.edu.au (B.K.); hyeatman@uow.edu.au (H.Y.); 2 School of Population Health, University of Auckland, Auckland 1072, New Zealand; boyd.swinburn@auckland.ac.nz; 3 School of Biosciences, Faculty of Health and Medical Sciences, Taylor’s University, Subang Jaya 47500, Malaysia; 4 Dietetics Program, Faculty of Health Sciences, Universiti Kebangsaan Malaysia, Kuala Lumpur 50300, Malaysia

**Keywords:** food, marketing, advertising, barrier, facilitator, policy

## Abstract

Unhealthy food marketing shapes children’s preference towards obesogenic foods. In Malaysia, policies regulating this food marketing were rated as poor compared to global standards, justifying the need to explore barriers and facilitators during policy development and implementation processes. The case study incorporated qualitative methods, including historical mapping, semi-structured interviews with key informants and a search of cited documents. Nine participants were interviewed, representing the Federal government (*n* = 5), food industry (*n* = 2) and civil society (*n* = 2). Even though the mandatory approach to government-led regulation of food marketing to children was the benchmark, more barriers than facilitators in the policy process led to industry self-regulations in Malaysia. Cited barriers were the lack of political will, industry resistance, complexity of legislation, technical challenges, and lack of resources, particularly professional skills. The adoption of industry self-regulation created further barriers to subsequent policy advancement. These included implementer indifference (industry), lack of monitoring, poor stakeholder relations, and policy characteristics linked to weak criteria and voluntary uptake. These underlying barriers, together with a lack of sustained public health advocacy, exacerbated policy inertia. Key recommendations include strengthening pro-public health stakeholder partnerships, applying sustained efforts in policy advocacy to overcome policy inertia, and conducting monitoring for policy compliance and accountability. These form the key lessons for advocating policy reforms.

## 1. Introduction

Low- and middle-income countries (LMICs) are not immune to obesity and non-communicable diseases (NCDs) [1,2]. In Malaysia, an upper-middle income country, half of its adult population are either overweight or obese, with an alarming prevalence of hypercholesteremia (38.1%), hypertension (30.0%) and diabetes (18.3%) [3,4]. Dietary risks rank as the top contributor to the burden of diseases in Malaysia, accounting for 13–14% of disability-adjusted life years between 1990 and 2017 [5].

Rising sales volume of ultra-processed products pose dietary risks for obesity and diet-related NCDs in LMICs [6,7,8,9]. Their high consumption is attributed to being cheap, convenient, palatable and heavily marketed [10]. Advertising of ultra-processed foods such as sugar-sweetened beverages, sweet snacks, fast foods and savoury snacks is dominant on Malaysian children’s popular television channels [11]. Exposure of children to such advertisements increases awareness of promoted foods and brands, establish food/brand attitudes and preferences, as well as foster purchase intent and consumption of the promoted products [12,13,14,15,16,17].

An effective policy is crucial to reduce children’s exposure to unhealthy food marketing [18,19]. Enacting government-led regulatory approaches to protect children up to 18 years old is desirable and this action aligns with the *United Nations Convention on the Rights of the Child* [18,20]. In 2018, only 30% of 142 WHO members had instituted food marketing regulations, with most offering limited protection only up to the age 12 or 13 years [21]. In the WHO Western Pacific Region, of which Malaysia is a member state, food marketing policies are mostly voluntary (5/31) or non-existent (17/31) [22]. Malaysia chose to adopt industry self-regulatory food marketing policies in 2008. The *Guideline on the Advertising and Nutrition Information Labelling of Fast Foods* (henceforth termed the ‘Fast Food Advertising Guideline’) selectively restricts fast food advertising during children’s programmes (e.g., cartoons) when ≥4% of television viewing audience is children aged 4 to 9 years [23]. The *Responsible Advertising to Children Initiative* (henceforth termed the ‘Pledge’), that mainly applies to retail foods, sets commitments for signatory companies on responsible marketing to children [24]. The *Pledge* is applicable to all broadcast periods when ≥35% of the audience are under 12 years of age. It also includes a minimal criterion for children’s settings with the signatory pledges to support for “no communication related to products in primary schools except where specifically requested by, or agreed with the school administration for educational or informational purposes” [24] (p. 1).

In performing the Malaysian Food-Environment Policy Index (Food-EPI) analysis, local public health experts considered the existing food marketing policies to be relatively weak [25,26], in comparison to the strong and enforceable legal frameworks available in Chile and South Korea [27]. Specifically, the experts gave low ratings to the implementation of policies to restrict the commercial promotion of unhealthy foods in children’s settings and on broadcast media [25]. Galbraith-Emami and Lobstein [28] warned that industry pledges have been ineffective in reducing children’s exposure to unhealthy food marketing, mostly due to their weak policy definitions and voluntary uptake.

In the last decade, countries such as Chile, Portugal and South Korea implemented mandatory regulations to reduce the unhealthy food marketing to children [27]. In contrast, the Malaysian Food-EPI finding on the poor implementation of food marketing policies triggered this case study to scrutinise “What are the barriers and facilitators during the policy processes to restrict unhealthy food marketing exposure to children?” Our case study aimed firstly to produce a historical map of local events and international directions up to 2017, and secondly to examine barriers and facilitators as determinants of policy processes. Evidence collected will contribute to advancing better policy understanding for the creation of healthy food environments from the perspective of an upper-middle-income country. Lessons gained from this case study should also guide policy entrepreneurs to reform future initiatives with a focus to improve public health nutrition.

## 2. Materials and Methods

A case study approach was applied to investigate the policy processes relating to food marketing to children in Malaysia. Ethics approvals were granted by the Research Ethics Committee of the National University of Malaysia (UKM PP1/111/8/JEP-2016-394); the Social Science Human Research Ethics Committee of the University of Wollongong (HE16/297); and the Medical Research and Ethics Committee, Ministry of Health Malaysia (NMRR-17-195-34142 (IIR)). Recruited participants to the case study included only those who returned consent forms.

### 2.1. Study Design

We adopted qualitative research methods to evaluate the research question. Semi-structured interviews were conducted along with collating publicly available information that was cited by the interviewed participants. Additionally, we performed a historical mapping of local events that related to food marketing up to 2017, along with aligning international directions on food marketing. This mapping of events contributed towards comprehending the policy processes in Malaysia. A guide used in conducting the interviews was based on an integrated theoretical framework to design and collect relevant information related to the policy processes. This framework comprised key components of the Advocacy Coalition Framework [29], the Model of Agenda Building [30] and the Theory of Coalition Structuring [31,32]. The interview guide has been described in another case study [33] and included as Supplementary Material S1.

### 2.2. Data Collection

**Public sector engagement**. The historical mapping of events enabled identification of the relevant government agencies involved in the policy processes. In July 2017, the researchers engaged with these agencies to access official government documents but the request was not approved under the *Malaysian Official Secrets Act 1972* [34]. However, stakeholders at these agencies agreed to review the preliminary historical map, as well as assign specific reference persons to be interviewed. Accordingly, the *Code of Ethics for the Marketing of Infant Foods and Related Products* [35], which applied to infants and toddlers <36 months was suggested by the stakeholders to be excluded from this case study. The rationale for this exclusion was that the policy processes involved a different timeline, triggers and stakeholders compared with the *Fast Food Advertising Guideline* and the *Pledge*.

**Recruitment process and selection criteria**. The engaged agencies nominated relevant government officials to be invited to semi-structured interviews, forming the first tier to be interviewed. Using a snowball sampling method [36,37], other public sector colleagues involved in the policy processes, and key stakeholders from industry and civil society, were identified. Eligibility criteria for participation were at least five years of work experience in a related field of expertise, agreement to declare conflicts of interest and granting permission to audio-record the interview.

Nineteen potential participants (government = 9; industry = 4; civil society = 6, included academia, non-profit organisations and professional organisations) were identified for the interview and formally invited, but only nine persons agreed to participate. Seven who refused consent gave reasons, such as being critically ill or lacking knowledge of the policy processes related to the investigated case. Three people did not respond to the invitation. Consenting candidates underwent a face-to-face semi-structured interview conducted by a research team member (SHN), according to their convenient venue and time. The interviews were conducted between June 2018 and February 2019.

**Protocol during the interview session**. Participants signed informed consent and provided their biographical information before been interviewed. The spoken language during the interview was English. Chronological events of the case were first presented to the participants at the interview session to simulate memory mapping [38]. Subsequent conversations were audio-recorded. Open-ended questions from the interviewer probed relevant information relating to the policy processes, using the oral history approach [39]. Participant feedback on how to improve the policy on food marketing was also obtained. For non-industry participants, additional questions specific to the food industry’s corporate political activities [40] were probed, such as lobbying, donation or sponsorship, partnership with stakeholders and applying legal action to impact the policy processes. Upon interview completion, participants’ feedback was invited on the significance of policy monitoring related to the case study. Potential key informants and publicly available information related to the case were sought from the participants. Fieldnotes complemented the interpretation of the recording.

### 2.3. Data Analysis

Data transcription was performed verbatim (SHN) and vetted by a co-researcher (TK) to ensure data consistency. Upon participant verification of the transcripts (*n* = 7), amendments (*n* = 4) were made for clarity, or to remove potentially identifiable statements. After completing the verification process, thematic analysis was performed using NVivo 12 software (QSR International Pty Ltd, Chadstone, Australia) with application of the constant comparison approach as described by Leech and Onwuegbuzie [41]. Theme and sub-themes were explored in reference to an earlier systematic review performed by the research team to investigate factors impeding and facilitating food environment policies [42]. Information cited by the case study participants was accessed, which related to publications from international (e.g., WHO and *Consumers International* documents) and national (e.g., government publications, memorandum, bulletins, newspapers and web pages) agencies to improve data interpretation and confirm the chronological timeline for the historical mapping.

From the collated information, the research team drafted the preliminary findings. The draft was reviewed by the engaged government agencies (*n* = 10 officers) to ensure data validity and sufficiency. The review process confirmed data saturation and yielded only minor amendments.

## 3. Results

### 3.1. Participant Characteristics

The nine participants interviewed for this case represented stakeholders from the Federal government (*n* = 5), food industry (*n* = 2) and civil society (*n* = 2), with each contributing an average 58 minutes to the interview process. Mean age of the participants was 53.8 ± 11.4 years, with all holding university education, including Master’s (*n* = 4), Bachelor’s (*n* = 3) and Doctoral (*n* = 2) qualifications (see Supplementary Table S1). They had 25.6 ± 9.7 years of work experience, covering areas such as food regulations, policy development and implementation, nutrition and public health. Seven non-industry participants were interviewed separately for information on corporate political activities.

### 3.2. Historical Mapping of the Food Marketing Policy Processes

Upon viewing the historical mapping related to the food marketing policy processes, participants narrated circumstances leading to these events. These were obesity and the NCD burden [43,44,45], the growing workforce of women [46] and the fast food industry expansion [45] in Malaysia which triggered concerns about unhealthy food consumption. The first *National Plan of Action for Nutrition of Malaysia* (NPANM) 1996–2000 was recommended to regulate fast food advertisements [44], although no significant action ensued. International directions from the mid-2000 period such as the *WHO Global Strategy on Diet, Physical Activity and Health* [47] and emerging evidence on the impact of food marketing on children and youth [48,49,50] set the agenda for member states. Building from the international directions in Malaysia, NPANM II 2006–2015 favoured regulating food advertisements in the mass media [51]. In 2007, the *Fast Food Advertising Guideline* was launched [23,52], forming one of the food marketing policies in this case study.

International directions to regulate unhealthy food marketing to children between 2008 and 2013 were suggested by participants to stimulate national policy processes. Of importance were the WHO recommendations on food and non-alcoholic beverage marketing to children [18,53,54,55,56], along with the nutrient profile models for food categorisation [57], which were later incorporated as a global NCD prevention strategy and called for government-led criteria [58,59]. These activities underpinned the WHO regional directions since 2013 [60]. In tandem, *Consumers International* conducted a two-year campaign advocating responsible marketing to children [61]. In parallel, major food and non-alcoholic beverage companies voluntarily made global commitments and revised their pledge criteria [62,63]. In Malaysia, apart from NPANM II 2006–2015, the *National Strategic Plan for Non-Communicable Disease 2010–2014* gave additional inputs to regulate unhealthy food marketing to children [64]. The *National Food Safety and Nutrition Council* meetings agreed upon a ‘guideline’ for such advertising [65]. The *Federation of Malaysian Manufacturers* worked with the government to launch the *Pledge* in 2013 [24,66], as the second self-regulatory approach in Malaysia to control unhealthy food marketing to children. A similar adoption of pledges for self-regulation by industry also occurred in neighbouring countries, such as Thailand, the Philippines and Singapore [63].

The *Rome Declaration on Nutrition 2014* calling on governments to strengthen commitment to discourage unhealthy food marketing to children [67], stimulated policy actions within Malaysia. Civil society advocacy activities in Malaysia were cited by some participants to be in tandem with *Consumers International*’s advocacy [68,69]. Also, the WHO intensified action to establish regional nutrient profile models that would be appropriate [70,71,72,73,74] to assess the eligibility of foods and beverages marketed to children. Specific thresholds of energy and/or nutrients (e.g., total fat, saturated fats, *trans*-fat, added sugar, total sugars, non-sugar sweetener and/or sodium) were established based on region-specific food products. In 2015, Malaysia participated in the development of a nutrient profiling model mooted by the *WHO Regional Office for the Western Pacific* (WPRO) [74]. The WPRO covered food marketing to children in the action plan for malnutrition and NCDs prevention [75,76], provided technical support to guide members on food marketing policy [77,78,79] and fostered a resolution and regional action plan to protect children from unhealthy food marketing [80,81,82]. In Malaysia, the NPANM III 2016–2025 planned regulation to restrict unhealthy television food advertising to children, as well as outlined intentions to develop specific nutrition criteria and to ban unhealthy food marketing within 50m of school perimeters [83]. In 2017, the *Cabinet Committee for a Health-Promoting Environment*, chaired by the Deputy Prime Minister, discussed policy options to ban unhealthy advertising of foods and beverages with high fat, salt and sugar content [84].

**Figure 1:**
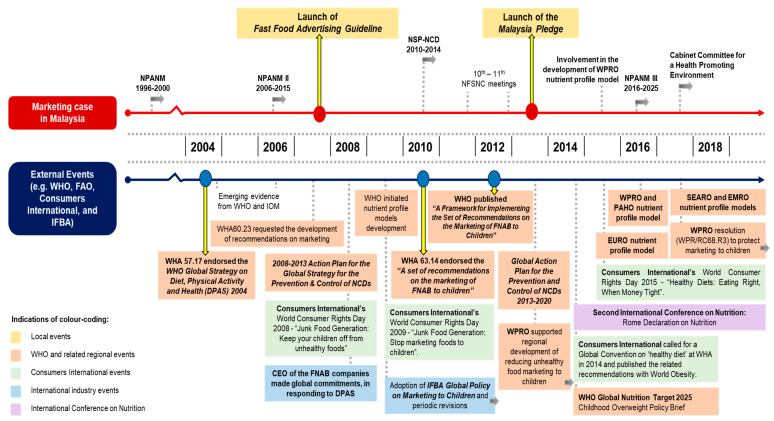
Historical mapping of food marketing policy processes in Malaysia. Abbreviations: CEO = Chief Executive Officer; DPAS = WHO Global Strategy on Diet, Physical Activity and Health; EMRO = WHO Regional Office for the Eastern Mediterranean; EURO = WHO Regional Office for Europe; FNAB = food and non-alcoholic beverages; IFBA = International Food & Beverage Alliance; IOM = Institute of Medicine; NCDs = non-communicable diseases; NFSNC = National Food Safety and Nutrition Council; NPANM = National Plan of Action for Nutrition of Malaysia; NSP-NCD = National Strategic Plan for Non-Communicable Diseases; PAHO = Pan American Health Organization; SEARO = WHO Regional Office for South-East Asia; WHA = World Health Assembly; WHO = World Health Organization; WPRO = WHO Regional Office for the Western Pacific.

The historical map of policy processes for the marketing case is presented in Figure 1, with a detailed description provided in Supplementary Material S2.

### 3.3. Key Themes

Thematic analysis of determinants forming barriers or facilitators to the policy processes revealed six emerging themes and relevant sub-themes that are listed in Table 1. The following section outlines participants’ feedback on the policy processes, described in accordance with the major themes and related sub-themes.

#### 3.3.1. Policy Commitment

Barriers identified in both policy development and implementation processes linked to policy commitment were ‘lack of resources’ and ‘lack of political will’. A ‘lack of sustained effort’ and ‘implementer characteristics’ were also viewed as barriers to policy implementation. Factors perceived to have facilitated policy processes were ‘resource availability or maximisation’ and ‘leadership’ from the health sector.

‘Lack of resources’ was acknowledged to hinder the policy processes by participants from the background of government and civil society. This barrier included a lack of professional skills in stakeholders relevant to food marketing policy, lack of funding for monitoring, as well as insufficient evidence to inform policy decisions. For instance:

*“The Ministry of Health would not be able to [either] monitor or enforce… not at the capacity to monitor… [hence] they [i.e., the advertising industry] took up the role….”*

(Government stakeholder on development and implementation).

‘Lack of political will’ to legislate towards restriction of unhealthy food marketing to children hindered policy progress. The following comments reflected this opinion:

*“I [first] ‘assumed’ it would be based on some kind of legislation... But, [later it was] the Pledge...”*

(Civil society stakeholder on development).

*“The Malaysian intention was [to] implement the resolution [of World Health Assembly (WHA) 63.14], but along the way, it became voluntary… I used the word “Executive Decision” … … the [authority] did not want to make any major shift… [despite] the scientific evidence is there… it is more on political will... There was no interest.”*

(Government stakeholder on development and implementation).

‘Implementer characteristics’ determined the urgency of policy adoption. For instance, small and medium enterprise (SME) businesses were less interested in implementing the *Pledge*, whilst companies that sold products with less healthy nutrition profiles also were reluctant to participate in the *Pledge*. The comment below reflected these views:

*“Not all companies have to be in… some can be quite sensitive… … we [SME] are doing more [on] reformulation first… we can join the Pledge, [but] after this.”*

(Industry stakeholder on implementation).

‘Lack of sustained effort’ in civil society advocacy was an impediment to policy advancement. This view was reflected by the following comments:

*“I did not see any NGO [non-government organisation] in Malaysia that is strong…”*

(Government stakeholder on implementation).

*“A major global issue [where the] Consumers International had a junk food generation campaign [and] World Consumer Rights Day… Apart from that, [no] sustained campaigns.”*

(Civil society stakeholder on implementation).

One participant provided an insider-view that a technical group within government from the health sector had begun working towards regulating unhealthy food marketing to children, as proposed in NPANM III 2016–2025. This facilitated ‘leadership’.

‘Resource availability or maximisation’ guided the policy processes, particularly referring to the WHO recommendations. A comment reflecting this view was:

*“There was a specific recommendation [from] the WHA’s Resolution. We had the guidance from the WHO documents… looking at whatever WHO is recommending... [like] ‘peak hours’…”*

(Government stakeholder on development).

#### 3.3.2. Policy Governance

Two sub-themes of policy governance, ‘complexity’ and ‘lack of monitoring’, were identified as hindering the policy processes.

‘Complexity’ of legislation to restrict unhealthy food marketing to children was acknowledged by participants from government. This mainly related to the marketing policy being difficult to align with existing policy framework and directions at the time. A comment reflecting these views was:

*“Do not have anywhere to park [the legislation] … same for Fast Food [Advertising Guideline] … under the Food Act [or Food Regulations], the mandate is more on the health hazard, food safety and also fraud... unhealthy food is not under [the purview of this] mandate... If the foods can be sold in the market… [it is] contradictory [to] restrict the selling of that foods [using the existing legislations] … [including the Ministry of Communication, the proposed legislation] also cannot park under their [regulations].”*

(Government stakeholder on development, implementation and future plans).

‘Lack of monitoring’ emerged as another critical barrier during the implementation of both the *Fast Food Advertising Guideline* and the *Pledge*. These policies were the responsibility of industry but without transparent reporting or accountability processes. As a consequence, the limited availability of monitoring data raised credibility issues. For instance:

*“No [government-led monitoring for the Fast Food Advertising Guideline] … … the Malaysia Pledge… it is an industry driven [monitoring].”*

(Government stakeholder on implementation).

*“… the [Fast Food Advertising] Guideline… nobody tells me, is it done or not… … [and also] the Pledge, I asked [industry group and] government, both sides... “Is it working or not?”. I do not see that [i.e., any compliance report being published], until today.”*

(Civil society stakeholder on implementation).

#### 3.3.3. External to Policy Organisation

‘Stakeholder relations’ was identified as a barrier to policy development and implementation. Specific to the *Pledge*, ‘stakeholder partnership or support’ was recognised as a facilitator during policy development.

Poor ‘stakeholder relations’ stemming from a lack of coordination, either within government sector or between policy makers and key stakeholders (e.g., broadcasters, advertisers), was considered to weaken public health interests and diminish policy outcomes. The lack of coordination was reflected by the following comment:

*“[Agency A] can do whatever... They do not engage us [Agency B] or industries per se... when [Agency A] chaired, suddenly, “It is a law”. I mean, where is the Committee? Who decided on that?”*

(Government stakeholder on development and implementation).

‘Stakeholder partnership or support’ from academia and professional organisations led to the development of the *Pledge*. Most participants considered this as a compromise solution to resolve temporary needs and local challenges, based on limited resources and capacity (e.g., lack of resources linked to professional capacity and guidelines for product classifications). Comments indicating these views were:

*“For a start… a few years, do that Pledge and see how it looks. [See if it is] difficult [to be] compliant...”*

(Civil society stakeholder on development).

*“It is based on the consensus meeting… [the stakeholders] agreed with 12 [years old for the Pledge] … in between the [fast food advertising] guideline up to 10 [years old and the] Children’s Act up to 14 [years old].”*

(Government stakeholder on development).

#### 3.3.4. Industry

For most participants, it was clear that the direction of the Malaysian food marketing policies linked to industry interests. Industry resistance to government regulations that aligned with WHO recommendations was evident which led towards industry preference for self-regulation.

At the time of developing the *Fast Food Advertising Guideline*, ‘industry resistance’ to regulation was prevalent, with the fast food industry seen to shift the blame for obesity and poor diets to other commercial food sectors. A participant cited that:

*“Initially, they [fast food industry] did not agree... their reason is why [are we] blaming them… in terms of obesity… maybe other factors… how about the mamak foods [i.e., local Tamil Muslim food sector] and all the other things?”*

(Government stakeholder on development).

With regard to development of the *Pledge*, further ‘industry resistance’ through information and messaging, policy substitution and constituency building were cited. Such practices were evident from the following comments:

Information and messaging strategy: *“I can only “assume” what has happened... but I do not have any evidence… [they are] working in the background… direct or just lobbying the [relevant] Ministries using their counter-arguments… very powerful lobbying by the industry.”*

(Government and civil society stakeholders on development).

Policy substitution strategy: *“[as] we [the government] could not implement an aggressive manner at that time… they [industries] came forward to make the [Pledge] proposal.”*

(Government stakeholder on development).

Constituency building strategy: *“… lobbying [the] professional associations… [where their] opinions are keen towards the industry.”*

(Government stakeholder on development).

Further probing of the relationship between the food industry and professional associations elucidated discrepancies in opinions between participants from different backgrounds, as evidenced from the comments below:

*“… if a company supports [professional organisation’s] activities… our views can be compromised. That’s definitely not so…”*

(Civil society stakeholder).

*versus*

*“No company will want to sponsor you without a return of investment... any sponsorship in cash or in-kind, it does have impact on bodies [to] behave towards the industries...”*

(Government stakeholder).

‘Industry engagement or support’ primarily related to development of the self-regulatory policies. Industry favouring the development of self-regulatory marketing policies in Malaysia was reflected in the following comment:

*“The Malaysia Pledge… industries were very much supportive… [some companies] had their counterparts in other countries [that were] also involved in [such a] Pledge. So, they moved [towards that].”*

(Industry stakeholder on development).

#### 3.3.5. Policy Specific Issue

‘Technical challenges’ and ‘policy characteristics’ emerged as two barrier sub-themes in policy development and implementation. In addition, the consequence of having ‘non-mandatory’ policies was recognised as a barrier during policy implementation due to lack of reporting and accountability requirements.

‘Technical challenges’ were highlighted in defining the policy scope. Despite new evidence emerged over time to guide policy actions, its compatibility to the local context was untested. For example, definitional issues and inadequate policy frameworks were raised. Comments manifesting these views included:

*“At that time… one of the questions is “what is fast food?”… there is no definition internationally… … [for the Pledge], “How [do you] categorise foods?... No guideline at that time … to combine [different nutrient] profiles from companies [was also] very challenging... Which [television] programmes [to be controlled]? How do [you] define children?”*

(Government and civil society stakeholders on development).

*“The nutrient profiling... the challenge is to set criteria…. Along the way, [we referred to] WHO reports... but, whether we can use [them] for Malaysian foods or not... There is still a question…”*

(Government stakeholder on future plans).

‘Policy characteristics’ that impeded the policy processes were loose technical criteria and the high costs of compliance monitoring. Specific to the *Pledge*, its ‘non-mandatory’ nature deterred the progress of policy implementation. These views were reflected by the following comments:

*“… this is not a law [for Fast Food Advertising Guideline], but like CSR [Corporate Social Responsibility] … no law [to] take them to court [for non-compliance] … … [Likewise]… no confidence in Pledge… sceptical about self-regulation.”*

(Government and civil society stakeholders on development).

*“[Fast food advertising] guideline… too limited [in] scope [and] very small niche… … [the Pledge] is voluntary… [causing] a double standard... not many industries [participated and] those who signed, you need to be good boys... no urgency in the implementation of the Pledge with other industries... … That’s why it is quite hard... to push for more than what [is] being written [and] to control.”*

(Government stakeholder on implementation).

#### 3.3.6. Opportunistic Advantages

‘Policy windows’ were perceived to foster the development of food marketing policies. International directions and top-level commitments coupled with local events concerning obesity and overweight rates, stimulated the national agenda to explore other policy options such as to restrict unhealthy food marketing to children. For instance:

*“WHO Guideline—restriction on marketing of foods to children… was the drive, and also Malaysia wanted to fulfil that pledge to WHO…”*

(Civil society stakeholder on development).

### 3.4. Recommendations

Participants at the end of the interview provided ideas to progress and improve food marketing policy processes in Malaysia. Table 2 summarises these ideas reflecting seven recommendations drawn from the interview information on barriers and facilitators in the policy processes.

## 4. Discussion

This case study provided an in-depth assessment of the aetiology of food marketing policies in Malaysia up to 2017 that ideally benchmarked to WHO recommendations, which enabled us to identify key barriers to the policy processes. From the mid-1990s, the Malaysian government expressed concerns [44] about the impact of television fast food marketing on children, and accordingly introduced the *Fast Food Advertising Guideline*. The WHO timeline, as described in the historical mapping for the case study, guided subsequent policy processes on unhealthy food marketing to children in Malaysia. However, Malaysia adopted the self-regulatory pathway to fulfilling the country’s commitment to WHA 63.14 [65], instead of implementing the best practice of government-led legislations. Of note, the timeline of the ensuing Malaysia *Pledge* aligned to the global position of the food industry’s agenda on promoting self-regulation in food marketing [62,63].

In general, case study participants acknowledged more barriers than facilitators (7 *versus* 4) during the development of food marketing policies, as well as when implementing these self-regulatory policies and progressing towards future plans (10 barriers *versus* 2 facilitators). Thus, it was unsurprising that Malaysia, an upper-middle-income country, encountered major obstacles in enacting a comprehensive policy to reduce unhealthy food marketing. Most of the barriers and facilitators identified in this Malaysian case study are comparable to those encountered by other LMICs when developing and implementing food environment policies, as evidenced in a recent systematic review [42] (*under review*). ‘Stakeholder relations’ posed a critical barrier to both policy development and implementation, which we identified in this case study but not prevalent in the previous studies relating to food marketing policies [36,37,85]. Another case study covering mandatory nutrition labelling in Malaysia, also identified ‘stakeholder relations’ as a barrier [33]. Therefore, with reference to Malaysia, systemic influences to progress local policy reforms are required to halt inherent policy inertia.

The case study highlights that a self-regulation policy agenda likely encourages inefficiency or a motive of intended stalling in policy implementation because of the lack of compulsion to adopt commitments by the food industry. Self-regulation although benefiting the goal of no change to the *status quo* of the industry, will diametrically oppose the public health goal of protecting children. This issue is well cited in literature, with voluntary participation in industry self-regulation schemes often linked to policy ineffectiveness, highlighting the need for better accountability, non-biased evaluation and sanctioning mechanisms [86,87]. Prior to this study, the WPRO [77] had already identified inherent limitations with the Malaysia *Pledge*, arising from the lack of standardised nutrition criteria, unreliable self-monitoring, unclear commitments and its voluntary adoption by the signatories. This is consistent with the case study participants’ reservations about the likely lack of impact of self-regulation, due to its slow progress, weak criteria and lack of a comprehensive monitoring framework. As expected of ‘non-mandatory’ policies, these challenges further weaken policy implementation and/or future plans. Several studies have identified concerns specific to non-mandatory policies in Malaysia. These concerns include children’s exposures to unhealthy food marketing and their vulnerability to its power [11,15,88], as well as poor media literacy education to moderate consumption of advertised foods [89]. Furthermore, the majority of the prominent food companies in Malaysia had poorer food industry commitments and disclosures related to promotion practices, compared to other countries [90,91]. Such local evidence highlights the need to enact stricter and comprehensive regulatory food marketing policies as a priority.

A positive development identified during the interviews was the establishment of a technical group within government to progress plans as outlined in the NPANM III 2016–2025 [83]. However, there was a 12-year transition period from initial documentation of the agenda in NPANM II to materialise support for regulating food marketing in 2006 [51], which essentially reflected a very long gestation period. Although advocacy to regulate unhealthy food marketing was observed in Malaysia [92,93,94], there was no reported discernible impact on policy outcomes with implementation gaps [25,90], implying lack of influence of such advocacy. In Chile, the transition period was about 13 years between policy formulation and the application of a new food act to regulate unhealthy food advertising and introduce warning labels on food packaging [95]. Efforts in Chile were impacted by strong opposition from the food industry [95], a factor that also influenced policy development in Malaysia.

A keen observation of participants was that industry was more cooperative with policies carrying minimal negative implications for and maximum benefits to company revenues. This collaborative approach potentially integrated with corporate political activities such as lobbying, policy substitution and constituency building with professional associations, as understood from the case study interviews. It was interesting to note that opinion on corporate political activities varied amongst participants, depending on their professional background. The ability of these activities to impede food marketing policy processes have been observed in other countries. For instance, food industries in Mexico perceived legislation as a threat to profits and proposed a less restrictive self-regulatory code to govern promotion practices [37,96,97]. In the Philippines, lobbying and pressuring of policymakers to lift school food marketing policy have been reported [37]. Furthermore, the linking of corporate social responsibility activities to branding by food companies has been found to compromise policy processes that aim to reduce the impact of unhealthy food marketing to children [37,97]. In Malaysia, brand-linked corporate social responsibility activities by food companies prevail [90], further leading to potential risk to policy progress. Such industry actions warrant the establishment of mechanisms to mitigate conflicts of interest, in accordance with the principles outlined in the literature [98,99].

Complex issues in the regulatory framework to restrict unhealthy food marketing were acknowledged by case study participants, including the lack of regulations to govern unhealthy foods. Such issues also have been observed in Australia [85] and Thailand [36], especially when the policy is considered contradictory to dominant economic policies or has unclear jurisdictional responsibility. In Malaysia, the existing regulatory framework is limited to governing physical health hazards, food safety and fraud issues [100] and thus lacks a legal framework to take on challenges to regulate unhealthy food marketing to children. To change the *status quo* to a comprehensive regulatory framework, this requires strong and proactive leadership. Some case study participants suggested endorsement by the World Trade Organization of WHO recommendations would elevate a policy’s status and overcome competing policy considerations, which is a view that aligns with Swinburn et al. [10]. Resolving these challenging issues will be important for building a comprehensive regulatory framework to tackle unhealthy food marketing in both media and in children’s settings. At the time of the case study, participants put forth strategies to progress future plans, such as stakeholder collaboration, sustained monitoring and advocacy. Their recommendations resonate with the recent report, *Regional Action Framework on Protecting Children from the Harmful Impact of Food Marketing in the Western Pacific* [22], which highlights the effectiveness of building a multi-sectoral effort centred on common objectives to progress policy actions.

Another approach to breaking policy inertia is to maximise resources. Evidence and technical support from national and international resources may expedite the policy process. In the past, WHO regional offices supported Tonga to progress a food environment policy supporting the restriction of fatty meat imports [101], as well as Mexico [96] for food marketing regulations. Alternately, breaking the stagnation of policy inertia requires understanding the mindset of the key players in food marketing, and engaging food companies and the service providers such as advertising agencies and media without undermining public health interests. Indeed, case study participants recommended the need to understand and engage the entire food marketing ecosystem, as well as build an accountability system to hold stakeholders responsible for marketing to children.

A major strength of this case study is that barriers and facilitators inherent to the self-regulatory food marketing policy processes for an upper-middle income country such as Malaysia were identified. These indicators will serve as a reference to policy entrepreneurs in countries with a similar background to understand and develop relevant strategies to maximise opportunities to reduce harmful impacts of food marketing to children. A limitation of this case study is the small sample size of participants, which was expected as only a few stakeholders were involved in the policy processes in Malaysia. Other studies that have analysed policy processes have had similar sample sizes [97,102,103,104]. Despite having only nine contributors to this case study, they held seniority in professional experience with specialised knowledge in policy areas. A further limitation was the lack of access to critical government documents, which we overcame by engaging government agencies to review the preliminary findings. As the case study adopted a qualitative research approach with interviews, recall bias and representativeness issues may limit interpretation. However, data interpretation was improved by applying the integrated theoretical framework to probe relevant information from different participant backgrounds via semi-structured interviews and reached data saturation, coupled with a historical mapping and a search for publicly available information.

Overall, this case study contributes evidence related to self-regulatory food marketing policy processes, witnessing Malaysia’s transition from a high (before 2016) to very high (2016 onwards) human development index country [105] but it is still weak in civil society advocacy. The findings resonate with, and may be generalisable to other South East Asian countries, as well as most LMICs. The theoretical framework identified fundamental challenges expressed in the case study that influenced government policy actions, leading to the adoption of self-regulatory pledges and policy inertia. These challenges include: (1) the lack of strong political will; (2) corporate political activities related to the risk of revenue loss to industry; (3) low priority for this issue relating to resource capacity of non-industry stakeholders that lacking professional expertise, funding, evidence, technical knowledge and monitoring skills; (4) technical challenges to classify healthiness of food products in the period pre-empting the WHO nutrient profile models; (5) failure to integrate the mandatory approach into the existing regulation framework; and (6) lack of inter-agency coordination between health governing agencies, advertisers and broadcasters, as well as among advocates to demand policy changes. Challenges related to industry resistance, lack of political will and insufficient resources were also observed in Thailand, when implementing a policy to restrict unhealthy food advertising to children aged 3 to 12 years old [36].

This case study offers perspectives that could be further explored in future research. For instance, the relatively weak controls on unhealthy food marketing in Malaysia [25,90] may amplify the impacts of higher media and screen use in young people during the COVID-19 pandemic. Case study findings will provide a baseline data to stimulate further research, such as broadening the scope of stakeholder participation namely politicians, public health lawyers, parents, broadcasters and advertisers, which could lead towards enactment of a comprehensive regulatory framework. Health impact assessment favouring public health interest could be another future research area to support the justification for a mandatory regulatory amendment towards restricting unhealthy food marketing to children. The case study findings should also stimulate future comparative research from countries sharing similar policy backgrounds.

Participants highlighted recommendations to progress policy implementation, comprising stronger leadership, resources, inter-ministerial coordination, advocacy partnerships and accountability monitoring systems. Sisnowski et al. [106] indicated that establishing a coordinating agency for inter-ministerial collaboration enabled policy progress. The agency could develop common objectives across ministries, maximise resource sharing, build a strong collaboration with external organisations and increase accountability between agencies.

## 5. Conclusions

This case study synthesises evidence from stakeholders involved in policy processes of food marketing targeting children in Malaysia and policy inertia that impacts policy progress. The policy outcomes contributed to the development of a self-regulatory approach, whilst the policy processes faced more barriers than facilitators. Barriers that favoured self-regulation were poor political will and insufficient resources available to address industry opposition and overcome governance difficulties. Policy processes were impacted by lack of credible monitoring, policy specific issues and implementer characteristics. These factors will likely impact future policy success. Policy entrepreneurs should adopt the recommendations outlined in this case study and allocate sufficient resources, particularly in developing a transparent monitoring and evaluation systems. Resolving barriers in the policy processes should enable a comprehensive and effective policy enactment, protecting vulnerable children from unhealthy food marketing and guiding future policy actions.

## Figures and Tables

**Table 1:** Overview of thematic findings related to policy processes from interviews.

Theme	Nature	Sub-Theme	Policy Process	
			Development	Implementation/Future Plans
Policy commitment	Barrier	Lack of resources	√	√
	Barrier	Lack of political will	√	√
	Barrier	Implementer characteristics	X	√
	Barrier	Lack of sustained efforts	X	√
	Facilitator	Leadership	X	√
	Facilitator	Resource availability or maximisation	√	√
Policy governance	Barrier	Complexity	√	√
	Barrier	Lack of monitoring	X	√
External to policy organisation	Barrier	Stakeholder relations	√	√
	Facilitator	Stakeholder partnership or support	√	X
Industry	Barrier	Industry resistance	√	X
Policy specific issue	Barrier	Technical challenges	√	√
	Barrier	Policy characteristics	√	√
	Barrier	Non-mandatory	X	√
Opportunistic advantages	Facilitator	Policy window	√	X

Symbols: √ = identified; X = not identified.

**Table 2:** Recommendations for stakeholders to progress food marketing policy plan.

Recommendations	
1.	Strong and proactive leadership to guard against political stagnation and commercial interests, and balancing public health interest to combat the influence of unhealthy food marketing to children.
2.	World Trade Organization endorsement of WHO recommendations for the restriction of unhealthy food marketing to children.
3.	Resource maximisation, particularly in using credible scientific evidence and providing education to all policy stakeholders (e.g., SME, broadcasters, advertisers and public).
4.	A comprehensive regulatory framework including strict enforcement that links non-compliance to consequences.
5.	Strengthening inter-ministerial collaboration (e.g., find solutions for governance complexity, set common objectives) and engaging with the key external stakeholders of food marketing policies (e.g., broadcasters and advertisers).
6.	Mapping positions of NGO stakeholders with shared interests in restricting unhealthy food marketing to children and forming a pro-public health coalition with sustained advocacy actions.
7.	Integration of sustained and transparent monitoring and evaluation systems, with the involvement of civil society and academia that pose none conflicts of interest.

Abbreviations: NGO = non-government organisation; SME = small and medium-sized enterprises; WHO = World Health Organization.

## Data Availability

Not applicable as audio records could not be made publicly available to protect confidentiality of the participants as per the ethics requirements.

## Supplementary Materials

The following are available online at https://www.mdpi.com/article/10.3390/ijerph18189607/s1, Supplementary Material S1: Interview guide; Table S1: Participant profiles, Supplementary Material S2: Historical Mapping of Food Marketing Policies in Malaysia.
