# Supplementary material for: Policy Inertia on Regulating Food Marketing to Children: A Case Study of Malaysia
Source: Int J Environ Res Public Health. 2021 Sep 12;18(18):9607. doi: 10.3390/ijerph18189607 (PMC8472389; doi:10.3390/ijerph18189607)
Supplement: Supplementary file 1 [file ijerph-18-09607-s001.zip › 3. Table S1 Participant profiles.pdf]

Table S1 – Participant profiles

Background	Overall field of expertise	Education level (number of participants)	Year of experience (Mean±SD)	Number of participants contributed in the following section	
				Self-regulatory food marketing case	CPA
Government	Food regulations, policy, standard or strategy development, programme planning and implementation, public health, prevention and control of non-communicable diseases, nutrition label, front-of-pack labelling, nutrition, dietetics, and international collaboration or engagement for nutrition and related strategies.	Degree (n=1)<br>Master (n=4)<br>PhD (nil)	23.6±8.8	5	5
Food industry	Food regulatory affairs (including food regulations compliance and technical meetings involvement in industry association and other stakeholders), and product development (i.e. leads R&D team).	Degree (n=2)<br>Master (nil)<br>PhD (nil)	21.0±12.7	2	N/A
Civil society	Public health nutrition, nutrients in foods, food regulations and consumer behaviours.	Degree (nil)<br>Master (nil)<br>PhD (n=2)	35.0±7.1	2	2
Overall		Degree (n=3)<br>Master (n=4)<br>PhD (n=2)	25.6±9.7	9	7

Abbreviations: CPA = Corporate Political Activity; N/A = Not applicable; nil = zero; PhD = Doctor of Philosophy; R&D = Research and development; SD = Standard deviation

Notes:

1. For food industry participants, corporate political activities were excluded from the semi-structure interviews. Civil society comprised of representatives from academia and non-governmental organisations.
2. Participants might have more than one background. The research team assigned the most relevant background corresponding to their roles during the policy processes.
