# Supplementary material for: Policy Inertia on Regulating Food Marketing to Children: A Case Study of Malaysia
Source: Int J Environ Res Public Health. 2021 Sep 12;18(18):9607. doi: 10.3390/ijerph18189607 (PMC8472389; doi:10.3390/ijerph18189607)
Supplement: Supplementary file 1 [file ijerph-18-09607-s001.zip › 4. Supplementary Material 2 Historical Mapping [Full].pdf]

Supplementary Material S2

Historical Mapping of Food Marketing Policies in Malaysia

Three national concerns influenced the policy window leading to the national discussion on fast food consumption. These were the alarming rates of obesity and NCDs [1-3], the growing participation of women in economic development [4], and the rapid expansion of the Malaysian fast food industry throughout most of the 1990s [3]. Later, the Malaysian government's concern regarding fast food commercials on the broadcast media, was articulated in the *National Plan of Action for Nutrition of Malaysia* (NPANM) 1996-2000 with a recommendation to regulate these advertisements [2]. However, significant action did not occur during the NPANM I 1996-2000 period as observed by the participants.

Participants observed that external events of the mid-2000 period such as WHA's endorsement of *Global Strategy on Diet, Physical Activity and Health* [5] and emerging evidence on unhealthy food marketing to children [6-8] set the agenda for member states. In Malaysia, NPANM II 2006-2015 outlined an activity to support the regulation and enforcement of food advertisements on mass media [9]. Later, a guideline for the fast food advertising was launched in 2007 and implemented in 2008 [10-11]. Until 2009, no major local event was observed after the implementation of the fast food advertising guideline.

Further external events were then recognised by participants to contribute to the subsequent local agenda setting in Malaysia. For instance, WHO initiated development of recommendations for the marketing of food and non-alcoholic beverages (FNAB) to children in 2007, which became a strategy for NCD prevention and control [11-12]. Consumer International conducted a two-year advocacy

campaign with the theme 'Junk Food Generation' for the *World Consumer Rights Days* in 2008 and 2009 [14]. To align with this, major FNAB companies voluntarily made global commitments on responsible advertising to children [15], followed by their *International Food & Beverage Alliance (IFBA) Global Policy on Marketing to Children* in 2009 [16]. The IFBA's revised pledge criteria was applicable to children under 12 years of age, cover school settings (2010) and change media audience cut-off from 50% to 35% or more of the audience under 12 years of age (2011) [16]. It was in 2010 that the Malaysian government published the *National Strategic Plan for Non-Communicable Disease 2010-2014*. This plan outlined a potential role for the Ministry of Information, Communication, Arts and Culture to regulate unhealthy food advertising to children [17]. In the same year, the *WHO's Set of Recommendations on the Marketing of FNAB to Children* was endorsed at the WHA 63.14 [18-19], followed by its implementation framework published 2 years later [20].

In Malaysia, the *National Food Safety and Nutrition Council* (NFSNC) meeting became the highest platform to discuss food safety and nutrition issues of the country [21]. At the 10th NFSNC meeting, the Ministry of Health (MOH) consented to develop a 'guideline' to control FNAB marketing to children [22]. This was further reaffirmed during a dialogue with the food and drink industries in 2011, following several commitments made by industry stakeholders to adopt an active role in multi-sectoral partnerships with the MOH [22]. In 2012, the Malaysian government officially adopted the 'guideline' announced at the 11th NFSNC meeting which fulfilled the country's commitment to WHA 63.14 [23]. The 'guideline', also termed as the 'Malaysia Pledge' by the industry [24], was officially launched in 2013 by the *Federation of Malaysian Manufacturers Malaysian Food Manufacturing Group* (FMM MAFMAG). Prior to the Malaysia *Pledge*, regional countries such as Thailand, Philippines, and Singapore had implemented similar programmes [16]. During the same period, *WHO's Set of Recommendations on the Marketing of FNAB to Children* became integrated into the preventive action

plan for NCDs [25]. WPRO also initiated an informal consultation in 2013, as a regional action to strengthen effort to restrict unhealthy food marketing to children [26].

In 2014, the WHO specifically targeted the need to address exposure of food marketing to children, and through the *Global Nutrition Target 2025 Childhood Obesity Policy Brief* called for government-led criteria [27]. Governments reaffirmed the commitment to WHA 63.14 resolution on unhealthy food marketing to children at the *Second International Conference on Nutrition* [28]. The *Consumer International*'s advocacy campaign themed 'Healthy diet' between 2014 and 2015 also targeted unhealthy food marketing to children [29-30]. Consumer advocacy activities in Malaysia was also in tandem with *Consumer International*'s advocacy. No other local events in 2014 were cited by the participants.

Another external event was when WHO commenced the development of nutrient profile models in 2009 [31], in order to assess nutritional quality of foods for multiple uses, including banning unhealthy food marketing to children. WHO's regional activities gradually accelerated to support the member states. For example, the WHO published regional nutrient profile models for marketing between 2015 and 2017 [32-36], with Malaysia partisan to the development of the *Western Pacific Region Office* (WPRO) nutrient profile model [36]. WPRO also established action plans [37-38] and conducted a series of technical meetings and workshops related to reducing unhealthy food marketing to children [36,39-41]. The Malaysian government also set activities under the NPANM III 2016-2025, including to use regulation to restrict television advertising of unhealthy foods or beverages to children, develop specific nutrition criteria for monitoring the Pledge and ban unhealthy marketing within 50m of the perimeter of schools [42].

In 2017, the *Cabinet Committee for a Health-Promoting Environment* chairing by the Deputy Prime Minister discussed thirteen policy options, including the enforcement of banning the advertisements of unhealthy foods and beverages with high fat, salt, and sugar content [43]. During the same period, WPRO set a specific resolution (WPR/RC68.R3) to protect children from the harmful impact of food marketing [44], and in the subsequent years WPRO efforts continued to develop a regional action plan [45-46].

References:

1. Khor, G.L.; Gan, C.Y. Trends and dietary implications of some chronic non-communicable diseases in peninsular Malaysia. *Asia Pac J Clin Nutr.* **1992**, *1*, 159-168.
2. National Coordinating Committee on Food and Nutrition (NCCFN). *National Plan of Action for Nutrition of Malaysia (1996-2000)*; NCCFN, MOH: Kuala Lumpur, 1995.
3. Noor, M.I. The nutrition and health transition in Malaysia. *Public Health Nutr.* **2002**, *5*, 191-195.
4. Ahmad, A. Country Briefing Paper – Women in Malaysia. 1998. Available online: https://www.adb.org/sites/default/files/institutional-document/32552/women-malaysia.pdf (accessed on 13 January 2020).
5. World Health Organization (WHO). *Global Strategy on Diet, Physical Activity and Health*; WHO: Geneva, 2004.
6. Institute of Medicine (IOM). *Food marketing to children and youth: threat or opportunity?*; IOM: Washington, 2006.
7. World Health Organization (WHO*). Marketing Food to Children: The Global Regulatory Environment*; WHO: Geneva, 2004.

8. World Health Organization (WHO). *The Extent, Nature and Effects of Food Promotion to Children: A Review of the Evidence – Technical Paper Prepared for the World Health Organization*; WHO: Geneva, 2006.
9. National Coordinating Committee on Food and Nutrition (NCCFN). *National Plan of Action for Nutrition of Malaysia (2006-2015)*; NCCFN, MOH: Putrajaya, 2006.
10. Food Safety and Quality Division (FSQD). Garis Panduan Pengiklanan dan Pelabelan Maklumat Pemakanan Makanan Segera. n.d. Available online: http://fsq.moh.gov.my/v6/xs/dl.php?filename=320c4443c5c9b60b44dbbf3a76d264dd.pdf (accessed on 9 April 2019).
11. Tee, E.S. Keeping an eye on fast foods. The Star. 2008. Available online: https://www.thestar.com.my/lifestyle/health/2008/02/17/keeping-an-eye-on-fast-foods (accessed on 18 March 2020).
12. World Health Organization (WHO). *Sixtieth World Health Assembly (14-23 May 2007): Resolutions and Decisions Annexes*; WHO: Geneva, 2007.
13. World Health Organization (WHO). *2008-2013 Action Plan for the Global Strategy for the Prevention and Control of Noncommunicable Diseases*; WHO: Geneva, 2008.
14. Consumer Affairs Commission (CAC). World Consumer Rights Day Fact Sheet - Stemming the Upward Trend in Non-Communicable Diseases (NCDs) by Tackling Unhealthy Diets. 2015. Available online: https://www.consumeraffairsjamaica.gov.jm/portal/index.php?option=com_phocadownload&view=category&download=61:world-consumer-rights-day-2015-fact-sheet&id=5:consumer-advice&Itemid=311 (accessed on 13 January 2020).

15. International Food & Beverage Alliance (IFBA). A Global Commitment to Action on the Global Strategy on Diet, Physical Activity and Health (Letter). 2008. Available online: https://ifballiance.org/uploads/media/59de14cc5ce7f.pdf (accessed on 13 January 2020).
16. International Food & Beverage Alliance (IFBA). *Five Commitments in Five Years Review of Progress 2008 – 2013*; IFBA, 2014.
17. Ministry of Health (MOH). *National Strategic Plan for Non-communicable Disease (NSPNCD): Medium term strategic plan to further strengthen the cardiovascular diseases & diabetes prevention & control program in Malaysia (2010-2014)*; NCD Section, Disease Control Division, MOH: Putrajaya, 2010.
18. World Health Organization (WHO). *Sixty-third World Health Assembly (17-21 May 2010): Resolutions and Decisions Annexes*; WHO: Geneva, 2010.
19. World Health Organization (WHO). *Set of recommendations on the marketing of foods and non-alcoholic beverages to children*; WHO: Geneva, 2010.
20. World Health Organization (WHO). *A framework for implementing the set of recommendations on the marketing of foods and non-alcoholic beverages to children*; WHO: Geneva, 2012.
21. Food Safety and Quality Division (FSQD). FSQ Annual Report 2011 (English). 2011. Available online: http://fsq.moh.gov.my/v6/xs/dl.php?filename=9ed1311341a5aa346286fb7fe367d4cb.pdf (accessed on 14 January 2020).
22. Non-Communicable Disease Section. Annual Report 2011; NCD Section, MOH: Putrajaya, 2011.
23. Ministry of Health (MOH). *Annual Report 2012: Ministry of Health Malaysia*; MOH: Putrajaya, 2012.

24. International Food & Beverage Alliance (IFBA). Malaysian Food and Beverage Industry's "Responsible Advertising to Children" Initiative (The Malaysia Pledge). n.d. Available online: https://ifballiance.org/wp-content/uploads/2020/10/MALAYSIA_PLEDGE_FINAL_.pdf (accessed on 13 July 2021).
25. World Health Organization (WHO). *Global Action Plan for the Prevention and Control of Noncommunicable Diseases 2013-2020*; WHO: Geneva, 2013.
26. World Health Organization Regional Office for the Western Pacific (WPRO). Informal Consultation on Reducing the Harmful Impact on Children of Marketing Foods, Beverages, Tobacco and Alcohol, Manila, Philippines, 25-26 September 2013: meeting report. 2014. Available online: https://apps.who.int/iris/bitstream/handle/10665/208783/RS_2013_GE_42_PHL_eng.pdf?sequence=1&isAllowed=y (accessed on 13 January 2020).
27. World Health Organization (WHO). Global nutrition targets 2025: childhood overweight policy brief. 2014. Available online: https://apps.who.int/iris/rest/bitstreams/665596/retrieve (accessed on 18 March 2020).
28. Food and Agriculture Organization of the United Nations & World Health Organization (FAO & WHO). Second International Conference on Nutrition – Conference Outcome Document: Rome Declaration on Nutrition (Rome, 19-21 November 2014). 2014. Available online: http://www.fao.org/3/a-ml542e.pdf (accessed on 18 March 2020).
29. Consumers International & World Obesity. Recommendations towards a Global Convention to protect and promote healthy diets. 2014. Available online: https://www.consumersinternational.org/media/2211/recommendations-for-a-convention-on-healthy-diets-low-res-for-web.pdf (accessed on 11 January 2020).

30. Consumers International. World Consumer Rights Day 2015 – Healthy Diets. n.d. https://www.consumersinternational.org/what-we-do/world-consumer-rights-day/wcrd-2015-healthy-diets (accessed on 11 January 2020).
31. World Health Organization (WHO). *Nutrient Profiling – Report of a WHO/IASO Technical Meeting, London, United Kingdom 4-6 October 2010*; WHO: Geneva, 2011.
32. World Health Organization Regional Office for the East Mediterranean (EMRO). *Nutrient profile model for the marketing of food and non-alcoholic beverages to children in the WHO Eastern Mediterranean Region*; EMRO: Cairo, 2017.
33. World Health Organization Regional Office for Europe (EURO). *WHO Regional Office for Europe nutrient profile model*; EURO: Copenhagen, 2015.
34. Pan American Health Organization (PAHO). *Pan American Health Organization Nutrient Profile Model*; PAHO: Washington, 2016.
35. World Health Organization Regional Office for South-East Asia (SEARO). *WHO Nutrient Profile Model for South-East Asia Region*; SEARO: New Delhi, 2017.
36. World Health Organization Regional Office for the Western Pacific (WPRO). *WHO nutrient profile model for the Western Pacific Region: a tool to protect children from food marketing*; WPRO: Manila, 2016.
37. World Health Organization Regional Office for the Western Pacific (WPRO). *Western Pacific Regional Action Plan for the Prevention and Control of Noncommunicable Diseases*; WPRO: Manila, 2014.
38. World Health Organization Regional Office for the Western Pacific (WPRO). *Action Plan to Reduce the Double Burden of Malnutrition in the Western Pacific Region (2015-2020)*. WPRO: Manila, 2015.

39. World Health Organization Regional Office for the Western Pacific (WPRO). *Biregional Workshop on Restricting the Marketing of Foods and Non-alcoholic Beverages to Children in the Western Pacific and South-East Asia (1-4 December 2015: Kuala Lumpur, Malaysia)*; WPRO: Manila, 2016.
40. World Health Organization Regional Office for the Western Pacific (WPRO). *Technical Meeting on the Regional Adaptation of the WHO Nutrient Profile Model to the Western Pacific Region (19-21 October 2015: Manila, Philippines)*; WPRO: Manila, 2016.
41. World Health Organization Regional Office for the Western Pacific (WPRO). *Regional Workshop on Regulating the Marketing and Sale of Foods and Non-alcoholic beverages at Schools (1-3 June 2016: Manila, Philippines)*; WPRO: Manila, 2016.
42. National Coordinating Committee on Food and Nutrition (NCCFN). *National Plan of Action for Nutrition of Malaysia (NPANM III) 2016–2025*; NCCFN, MOH: Putrajaya, 2016.
43. Arumugam, T. Policies to promote a healthier nation. New Straits Times. 2017. Available online: https://www.nst.com.my/news/exclusive/2017/12/318178/policies-promote-healthier-nation (accessed on 18 March 2020).
44. World Health Organization Regional Office for the Western Pacific (WPRO). Resolution – Protecting Children from the Harmful Impact of Food Marketing (WPR/RC68.R3). 2017. Available online: https://iris.wpro.who.int/bitstream/handle/10665.1/13719/WPR-RC068-Res03-2017-en.pdf (accessed on 14 January 2020).
45. World Health Organization Regional Office for the Western Pacific (WPRO). *Experts Consultation to Inform the Development of a Draft Regional Action Framework on Protecting Children from the Harmful Impact of Food Marketing: 2020-2030 (5-7 December 2018: Manila, Philippines)*; WPRO: Manila, 2019.

46. World Health Organization Regional Office for the Western Pacific (WPRO). *Member States Consultation on the Draft Regional Action Framework on Protecting Children from the Harmful Impact of Food Marketing 2020-2030 (26-28 March 2019: Manila, Philippines)*; WPRO: Manila, 2019.
